# Bioaerosols from composting facilities—a review

**DOI:** 10.3389/fcimb.2014.00042

**Published:** 2014-04-04

**Authors:** Nathalie Wéry

**Affiliations:** INRA, UR0050, Laboratoire de Biotechnologie de l'EnvironnementNarbonne, France

**Keywords:** compost, bioaerosol, microbial diversity, impact on health, dispersal, molecular tools

## Abstract

Bioaerosols generated at composting plants are released during processes that involve the vigorous movement of material such as shredding, compost pile turning, or compost screening. Such bioaerosols are a cause of concern because of their potential impact on both occupational health and the public living in close proximity to such facilities. The biological hazards potentially associated with bioaerosol emissions from composting activities include fungi, bacteria, endotoxin, and 1-3 β-glucans. There is a major lack of knowledge concerning the dispersal of airborne microorganisms emitted by composting plants as well as the potential exposure of nearby residents. This is due in part to the difficulty of tracing specifically these microorganisms in air. In recent years, molecular tools have been used to develop new tracers which should help in risk assessments. This review summarizes current knowledge of microbial diversity in composting aerosols and of the associated risks to health. It also considers methodologies introduced recently to enhance understanding of bioaerosol dispersal, including new molecular indicators and modeling.

## Introduction

Composting is a method of waste management based on the biological degradation and stabilization of organic matter under aerobic conditions. It results in a sanitized and stabilized product rich in humic substances that can be used as fertilizer (Sykes et al., 2007). Large-scale composting has become a commonly used method worldwide for diverting agricultural waste, sewage sludge and other organic waste from landfills and incinerators. The degradation of organic matter is carried out by a complex and highly dynamic microflora containing Gram-positive and Gram-negative bacteria and fungi (Ishii et al., 2000; Ryckeboer et al., 2003; Hansgate et al., 2005). During the composting process, along with the microbial degradation of organic matter, the physico-chemical conditions pH, temperature and moisture content evolve and changes in microbial diversity are important. The intense microbial activity associated with degradation of easyly-degradable compounds leads to a rise in temperature at the beginning of the process. The fermentation phase is characterized by the degradation of organic matter by thermophilic species. It is followed by a maturing phase with degradation of cellulolytic and ligno-cellulosic compounds and humification reactions. The dynamics of microbial diversity during composting has been recently revealed by high-throughput sequencing (De Gannes et al., 2013).

Normal operations taking place at composting plants can be the source of nuisance or pollution involving odors, noise, dust, leachate, and bioaerosols (Sanchez-Monedero et al., 2005). The emission of bioaerosols during operational activities increases the concentration of microorganisms in the air by several orders of magnitude (Persoons et al., 2010; ADEME, 2012). The implications of the release of bioaerosols is especially significant for composting plants operating in the open because their bioaerosols are released directly into the surrounding environment without any pretreatment using biofilters or bioscrubbers. This paper focuses on data collected from large-scale composting operations with open-air windrow systems, which today remains the predominant composting technology. It gathers together recent findings on bioaerosols emitted from composting facilities in terms of microbial diversity, sanitary impact and dispersal beyond the site.

## Microbial diversity

The term “bioaerosol” encompasses all particles having a biological source that are in suspension in the air and includes microorganisms (bacteria, fungi, virus, protozoa, algae, pollen…) as well as biomolecules (toxins, debris from membranes…) (Sykes et al., 2011). Current knowledge on microbial diversity in aerosol from composting facilities is largely focused on bacteria and molds. For more than a decade, actinomycetes, *Aspergillus fumigatus*, and *Penicillium* sp. have been recognized as the dominant culturable micro-organisms in composting bioaerosols (Millner et al., 1980; Fischer et al., 1999; Hryhorczuk et al., 2001; Kampfer et al., 2002; Ryckeboer et al., 2003). However, cultivation-based techniques systematically underestimate the diversity of bioaerosols. Albrecht et al. (2007) showed that only 1.5–15.3% of airborne bacterial cells of a composting facility enumerated by direct counting formed countable colonies after incubation on TSA-agar. Recent culture-independent studies using sequencing of 16S rRNA and 18S rRNA gave some new data on the microbial diversity in composting aerosols. Tables 1, 2 present, respectively, the bacterial and fungal species that have been identified in composting bioaerosols using both culture-dependent and culture-independent approaches.

**Table 1:** **Dominant bacteria identified in aerosols from composting facilities using culture-dependent and culture independent techniques from Reinthaler et al. (1997), Le Goff et al. (2010), Bru-Adan et al. (2009), ADEME (2012), Pankhurst et al. (2012), and Betelli et al. (2013)**.

**Phyla**	**Genus**	**Species**	Technique^*^
**FIRMICUTES**			
	*Bacillus*	sp.	Cult., Seq.
		*B. subtilis, B. smithii, B. coagulans*	Seq.
	*Ureibacillus*	sp.	Seq.
		*U. koreensis*	Seq.
	*Geobacillus*	sp.	Seq.
		*G. thermodenitrificans, G. caldoxylosilyticus*	Seq.
	*Thermoactinomyces*	sp.	Seq.
		*T. intermedius, T. sacchari*	Seq.
		*T. thalpophilus*	Cult., Seq.
		*T. vulgaris*	Cult., qPCR
	*Planifilum*	sp.	Seq.
		*P. yunnanesis*	Seq.
	*Clostridium*	*C. peptidovorans*	Seq.
	*Symbiobacterium*	*S. thermophilum*	Seq.
	*Ammoniphilus*	sp.	Seq.
	*Streptococcus*	*S. sanguinis*	Seq.
	*Staphylococcus*	sp.	Cult.
		*S. epidermidis*	Cult.
**ACTINOBACTERIA**			
	*Saccharopolyspora*	*S. rectivirgula (syn: Faenia rectivirgula, Micropolyspora faeni)*	Cult., Seq.
		*S. hirsuta*	
	*Saccharomonospora*	sp.	Cult., Seq.
		*S. glauca, S. caesia*	Seq.
		*S. viridis*	Cult., Seq.
	*Thermomonospora*	sp.	Cult., Seq.
		*T. mesouviformis, T. chromogena*	Seq.
	*Thermobifida*	*T. fusca*	Seq.
	*Streptomyces*	sp.	Cult., Seq.
		*S. thermoviolaceus, S. cellulosae, S. thermoatroviridis*	Seq.
	*Corynebacterium*	sp.	Seq.
		*C. variabile, C. efficiens, C. glutamicum*	Seq.
	*Nocardiopsis*	*N. composta*	Seq.
	*Thermocrispum*	*T. agreste, T. municipale*	Seq.
	*Actinomadura*	*A. hallensis*	Seq.
	*Rhodococcus*	sp.	Seq.
	*Rothia*	sp.	Seq.
	*Arthrobacter*	sp.	Seq.
	*Microbacterium*	sp.	Seq.
	*Kutzneria*	sp.	Seq.
**ALPHAPROTEOBACTERIA**			
	*Sphingomonas*	sp.	Seq.
		*S. suberifaciens*	Seq.
	*Brevundimonas*	*B. nasdae*	Seq.
	*Mesorhizobium*	sp.	Seq.
	*Devosia*	sp.	Seq.
**GAMMAPROTEOBACTERIA**			
	*Pseudomonas*	sp.	Cult., Seq.
		*P. aeruginosa, P. fluorescens, P. oryzihabitans*	Cult.
	*Acinetobacter*	sp.	Seq.
		*A. calcoaceticus, A. lwoffii*	Seq.
	*Enhydrobacter*	*E. aerosaccus*	Seq.
	*Moraxella*	*M. osloensis*	Seq.
	*Enterobacter*	*E. cloacae*	Cult.
	*Pantoea*	*P. agglomerans*	Cult.
	*Klebsiella*	*K. oxytoca*	Cult.
	*Proteus*	*P. mirabilis*	Cult.
	*Xanthomonas*	*X. maltophila*	Cult.
	*Serratia*	*S. rubidea, S. marcescens*	Cult.
**BETAPROTEOBACTERIA**			
	*Delftia*	*D. acidovorans*	Seq.
	*Alcaligenes*	*A. faecalis*	Cult.
**BACTEROIDETES**			
	*Flavobacteriaceae*	sp.	Seq.
	*Taxeobacter*	sp.	Seq.

* Cult., culture; Seq., 16S rRNA sequencing; for rRNA sequencing data, the genus and species names are given for percentage of similarity above 95 and 97%, respectively. Only phylotypes with abundancy above 1% of the total number of sequences are presented.

**Table 2:** **Dominant fungi identified in aerosols from composting facilities using culture-dependent and culture independent techniques from Le Goff et al. (2010), Bru-Adan et al. (2009), and ADEME (2012)**.

**Phylum or subphylum**	**Genus**	**Species**	Technique^*^
**ASCOMYCOTA**			
	*Aspergillus*	sp.	Cult., Seq.
		*A. fumigatus*	Cult., Seq
		*A. versicolor*	Cult., Seq.
		*A. candidus*	Cult.
		*A. nidulans*	Cult.
		*A. niger*	Cult.
		*A. flavus*	Cult.
		*A.eburneo-cremeus*	Cult.
	*Penicillium*	sp.	Cult., Seq.
	*Eurotium*	sp.	Cult.
	*Thermomyces*	*T. lanuginosus*	Seq.
	*Clathrospora*	*C. diplospora*	Seq.
	*Illosporium*	*I. carneum*	Seq.
	*Microascus*	*M. cirrosus*	Seq.
	*Neurospora*	sp.	Seq.
	*Paraphaeosphaeria*	*P. nolinae*	Seq.
	*Pithia*	sp.	Seq.
	*Cladosporium*	sp.	Cult., Seq.
	*Marcelleina*	sp.	Seq.
	*Talaromyces*	*T. byssochlamydoides*	Seq.
	*Madurella*	*M. mycetomatis*	Seq.
	*Chalara*	*hyalina*	Seq.
	*Geotrichum*	*G. candidum*	Seq.
	*Pichia*	*P. guilliermondii*	Seq.
	*Phoma*	sp.	Seq.
		*P. herbarum*	Seq.
	*Ascolobus sp.*	sp.	Seq.
	*Anguillospora sp.*	*A. rubsecens*	Seq.
	*Trichoderma*	sp.	Cult.
	*Emericella*	sp.	Cult.
	*Tritirachium*	sp.	Cult.
	*Alternaria*	sp.	Cult.
	*Verticillium*	sp.	Cult.
	*Didymella*	sp.	Cult.
	*Candida*	sp.	Cult.
**BASIDIOMYCOTA**			
	*Dichostereum*	sp.	Cult., Seq.
	*Coprinus*	*C. comatus*	Cult., Seq.
	*Athelia*	*A. bombacina*	Cult., Seq.
	*Ustilago*	*U. hordei*	Seq.
	*Clitocybe*	*C. candicans*	Seq.
	*Filobasidium*	*F. globisporum*	Seq.
	*Sistotrema*	*S. sernanderi*	Seq.
	*Vuilleminia*	*V. comedens*	Seq.
	*Exidiopsis*	sp.	Seq.
	*Acanthophysium*	*A. cerussatum*	Seq.
	*Boletellus*	*B. projectellus*	Seq.
	*Exidiopsis*	sp.	Seq.
	*Peniophora*	*P. nuda*	Seq.
	*Itersonilia*	*I. perplexans*	Seq.
	*Filobasidium*	sp.	Seq.
	*Perenniporia*	*P. subacida*	Seq.
	*Botryobasidium*	*B. subcoronatum*	Seq.
	*Dioszegia*	*D. aurantiaca*	Seq.
	*Coleosporium*	*C. asterum*	Seq.
	*Rhodotorula*	*R. minuta*	Seq.
	*Classicula*	*C. fluitans*	Seq.
	*Sporobolomyces*	sp.	Seq.
	*Rhodotorella*	sp.	Cult.
**MUCOROMYCOTINA**			
	*Mucor*	*M. plumbeus*	Cult., Seq.
	*Absidia*	*A. corymbifera*	Cult., Seq.
	*Pilobolus*	*P. phaerosporus*	Seq.
	*Rhizopus*	sp.	Cult.
	*Circinella*	*C. umbellata*	Seq.
**ENTOMOPHTHOROMYCOTINA**			
	*Furia*	*F. ithacensis*	Seq.
**ZYGOMYCETES**			
	*Conidiobolus*	*C. thromboides*	Seq.
	*Pandora*	*P. neoaphidis*	Seq.
	*Rhizomucor*	*R. miehei*	Seq.

* Cult., culture; Seq., 18S rRNA sequencing; for rRNA sequencing data, only phylotypes with abundancy above 1% of the total number of sequences are presented.

In two studies on aerosols collected during the turning of composting piles in the thermophilic phase (Le Goff et al., 2010) and during the screening of matured compost (Bru-Adan et al., 2009), *Fimicutes* and *Actinobacteria* were the two dominant bacterial phyla. From sequencing data present in public databases, it appears that *Firmicutes*, *Proteobacteria*, and *Bacteroidetes* are more dominant in compost than are *Actinobacteria*. In particular, the percentage of *Bacteroidetes* is much higher in compost than in composting bioaerosols. The selection of sporulating species during aerosolization may explain the dominance of *Firmicutes* and *Actinobacteria*. *Actinobacteria*, *Thermoactinomyces* sp. and *Bacillus* sp., in fact produce resistant spores that spread widely. Nielsen et al. (1997) analyzed the concentration of micro-organisms in bioaerosols related to the concentration in bulk samples of compost from household waste. They found that actinomycetes or their spores were particularly prone to becoming airborne (Nielsen et al., 1995). Using PLFA (PhosphoLipid Fatty Acid analysis), PCR-DGGE (Denaturing Gradient Gel Electrophoresis) and pyrosequencing, Pankhurst et al. (2012) have shown the influence that green-waste composting has on the on-site and downwind airborne microbial communities. They found that in some cases, gamma-*Proteobacteria* (*Pseudomonas, Acinetobacter*) can also dominate bioaerosols emitted by composting platforms. At the genus level, these studies confirmed the high representativity in bioaerosols of the following species which were already known as major components of compost microflora (Song et al., 2001; Steger et al., 2007): *Aspergillus*, *Penicillium*, *Bacillus*, *Thermoactinomyces*, *Thermobifida*, *Saccharomonospora*, and *Saccharopolyspora*. The studies provided interesting new data concerning the importance of the fungus *Thermomyces lanuginosus* and of the bacteria *Geobacillus* and *Planifilum* in composting bioaerosols. They also showed that thermophilic species were strongly represented, even in mature compost (34% of the total number of bacterial sequences in the study by Bru-Adan et al., 2009).

Concerning fungi, the samples collected during the thermophilic phase by Le Goff et al. (2010) were dominated by Ascomycota (*Thermomyces lanuginosus*, *Aspergillus*, *Penicillium*…) whereas the air sample collected during the screening of more matured compost mainly contained representatives of the *Basidiomycetes* group (59% of the sequences), although sequences closely related to *Aspergillus* were also recovered (9% of the sequences). The potential changes in the microbial diversity of composting bioaerosols during the process still remain to be better characterized. Further studies are also needed to explain the differences recorded between diversity in compost and diversity in the associated aerosols (enrichment in sporulating species). Finally, despite their potential impact on health, data on the presence and dispersal of virus or eucaryotes (amoeba, algae…) in composting aerosols are scarce. Conza et al. (2013) have recently demonstrated the presence of amoebae in composting aerosols. In molecular inventories based on 18S rRNA sequencing, sequences from algae and protozoa were obtained (Bru-Adan et al., 2009; Le Goff et al., 2010).

## Impact on health of the exposure to aerosols emitted from compost

Some pathogenic organisms (bacteria, viruses, and parasites) are present in raw materials and composts, notably pathogens of enteric origin in sludge from municipal sewage plants or animal waste, but such pathogens are rapidly inactivated by heat during the composting process. The main identified risks of infection from composting bioaerosols are represented by opportunistic micro-organisms, especially molds which can take advantage of deterioration in the immune system. Prolonged exposure to *Aspergillus fumigatus*, an opportunistic fungal pathogen, may cause invasive aspergillosis in immuno-compromised individuals. (Shen et al., 2004; Taha et al., 2006). Rare cases of invasive aspergillosis have been described among people exposed to dusts originating in decomposing vegetable matter (ADEME, 2012). However, data in the literature does not indicate an excess of severe infectious illness among compost workers. The main effects of exposure to composting aerosols are on respiratory health; these include organic dust toxic syndrome, extrinsic allergic alveolitis (EAA), allergic rhinitis, asthma, upper airway irritation and mucous membrane irritation (Swan et al., 2003; Sykes et al., 2007). *A. fumigatus* and thermophilic actinomycetes (*Thermoactinomyces vulgaris, Saccharopolyspora rectivirgula*) are implicated in hypersensitivity-induced pneumonitis and other allergic reactions such as alveolitis or bronchial asthma (Lacey and Crook, 1988; Dutkiewicz et al., 1994; Poulsen et al., 1995; Kampfer et al., 2002; Albrecht et al., 2008). In addition to these micro-organisms, certain biological agents can also affect human health: endotoxins, components of the cell wall of Gram-negative bacteria, peptidoglycans in the wall of Gram-positive bacteria, the β (1-3)-D-glucans in the cell wall of molds and the mycotoxins (Sykes et al., 2011). The main pathway leading to exposure is by inhalation of particles which reach the respiratory system. Particle deposition in lungs is closely related to their size. Many of the bioaerosol particles emitted by compost are very fine and can reach down the pulmonary alveoli (Chiang et al., 2003; Byeon et al., 2008). The size of spores of molds colonizing compost (*Aspergillus*, *Penicillium*) is below 3 μm (Madelin and Johnson, 1992) and the one of thermophilic actinomycetes is around 1 μm (Reponen et al., 1998).

Over the last 5 years, more knowledge has been acquired on the relevance of *Saccharopolyspora rectivirgula* and of *Legionella* species in aerosols from composting. *Saccharopolyspora rectivirgula* is often found in environments of agricultural production where the classic form of EAA (“farmer's lung disease”) is common. Schäfer et al. (2013) showed that high concentrations of airborne *S. rectivirgula* were to be found in composting plants at levels similar to those found in agricultural production. Using quantitative real-time polymerase chain reaction (PCR), they detected *S. rectivirgula* in 85% of the 124 aerosols sampled at 31 different composting plants. Estimated concentrations ranged between 1.2 × 10^2^ and 1.5 × 10^7^ cell counts/m^3^. Compost is also one of the recognized reservoirs of *Legionella*. One recent study has reported the presence of *L. pneumophila* and *L. bozemanii* and of free-living amoebae in compost and shown that the bioaerosols developed from 3 of the 4 composting facilities analyzed contain *L. pneumophila* (Conza et al., 2013). However, a survey of the seroprevalence of anti- *Legionella pneumophila* antibodies among workers composting sludge did not show a significant rise when compared to the non-exposed group (Clark et al., 1984).

The association between exposure to composting bioaerosol and adverse health effects has been demonstrated for compost workers (Herr et al., 2003; Bünger et al., 2007). According to Schlosser et al. (2009), the mean personal exposure levels to dust, bacteria, molds and endotoxins are fully consistent with the occurrence of inflammatory and allergic respiratory outcomes among workers. Certain studies have reported high levels of immunoglobulins in the blood of workers which suggests a high level of exposure leading to stimulation of the immune system (Clark et al., 1984; Beffa et al., 1998; Bünger et al., 2000, 2007). In a cross-sectional study, Van Kampen et al. (2012) investigated work-related symptoms and diseases of 190 currently-exposed compost workers, 59 former compost workers and 38 unexposed control subjects. Compared to controls, compost workers suffered more often from cough and irritation of the eyes in terms of mucosal membrane irritation. Former compost workers reported similar work-related complaints but these symptoms improved when exposure to bioaerosols ceased. In contrast, cough and dyspnea persisted, indicating a chronic process. There was no higher frequency of mold sensitization in the group of compost workers compared to controls, which, according to the authors, may be an indication of a healthy worker survivor effect.

Sykes et al. (2011) recommended that consideration be given to robust approaches to ensure dust suppression at source and that employees' exposures to organic dust are reduced as far as possible when waste is being agitated. Shredder and siever adjustments, sampling at the core of windrows in the turning phase, cleaning and maintenance of aeration/composting containers were found as producing the highest bioaerosols ambient concentrations by Persoons et al. (2010). Engineered measured such as water sprays, negative aeration systems or biofilters did not prevent on-site bioaerosol emissions. Composting in enclosed units prevent bioaerosol dispersal in the environment but is likely to increase occupational exposures.

Concerning nearby residents of composting plants, some epidemiological studies have found no relationship between respiratory symptoms and place of residence (Cobb et al., 1995), nor with the concentration of *Aspergillus fumigatus* (Browne et al., 2001). Others, in contrast, have shown that residents living within 150–200 m of a composting plant were affected, suffering from irritative respiratory complaints similar to mucous membrane irritation and from excessive tiredness (Herr et al., 2003).

## Dispersal of composting aerosols in the surroundings

The risk assessments undertaken to date have relied on air dispersion studies to estimate downwind concentrations of bioaerosols and to permit comparisons with data measured upwind or at background locations (Taha et al., 2006). Bioaerosol concentrations decrease rapidly with distance from their source and it becomes difficult to verify that measurements at a distance are related to a specific activity rather than to other non-compost sources (Taha et al., 2005).

The airborne microorganisms usually monitored in composting aerosols are cultivable bacteria and fungi (mesophilic and/or thermophilic) (Heida et al., 1995; Van Tongeren et al., 1997), Gram-negative bacteria or more definite microbial taxons such as *Aspergillus fumigatus* and actinomycetes (Millner et al., 1980; Gumonski et al., 1992; Darragh et al., 1997; Fischer et al., 1999; Hryhorczuk et al., 2001; Kampfer et al., 2002; Sanchez-Monedero and Stentiford, 2003; Sanchez-Monedero et al., 2005; Taha et al., 2006; Albrecht et al., 2007; Fischer et al., 2008; Schlosser et al., 2009; Pankhurst et al., 2011).

Thermophilic actinomycetes such as *Thermoactinomyces* and *Saccharomonospora* and thermotolerant microfungi have been put forward as potential indicators because they are rare in natural environments due to their thermotolerant or obligatory thermophilic characteristics. Their concentrations in air samples in the surroundings of composting plants are indeed higher than in background samples (Kampfer et al., 2002; Neef et al., 2003; Swan et al., 2003; Albrecht et al., 2008; Fischer et al., 2008). *Aspergillus fumigatus* is common in the environment but its concentration increases when there are sources of self-heating materials. For some authors, therefore, dominance of *Aspergillus fumigatus* in the downwind vicinity of a composting plant is an indication of the release of emissions from the plant (Recer et al., 2001; Taha et al., 2006; Albrecht et al., 2008; Pankhurst et al., 2011). The United Kingdom Composting Association has proposed a procedure for monitoring bioaerosols, based on the monitoring of two airborne groups, *Aspergillus fumigatus* and total mesophilic bacteria, at different upwind and downwind locations at a composting plant (Environment Agency, 2010).

Most studies on composting bioaerosols have been carried out using culture. However, the culturability of bacteria occurring in bioaerosols is low (Albrecht et al., 2007). Furthermore, culture techniques may underestimate the exposure to some composting bioaerosols; this is especially true for biological agents other than viable cells: endotoxins, mycotoxines, β (1-3)-D-glucans. In contrast to culture techniques, qPCR targeting DNA will not underestimate bioaerosol concentration. It is sensitive and robust, and is used widely for monitoring microoganisms in other environments (soil, water) (Peccia and Hernandez, 2006). Recently, thermophilic species from compost have been quantified by qPCR in order to monitor composting aerosols. Le Goff et al. (2010, 2011, 2012) used data obtained from molecular inventories to identify new indicators affiliated to *Saccharopolyspora rectivirgula*, to the *Thermoactinomycetaceae* and to the fungus *Thermomyces lanuginosus*. Schäfer et al. (2011, 2013) used qPCR to monitor *S. rectivirgula* in composting aerosols. Betelli et al. (2013) developed a system for monitoring *Thermoactinomyces vulgaris* as a basis for a standardized method for quantifying worker exposure to bioaerosols at composting facilities. To evaluate the exposure and the dispersal of composting bioaerosols, it is necessary to know their background concentrations in air from unaffected areas. Most studies have used concentrations measured upwind of the composting site with respect to the dominant wind. Table 3 gathers the microbial groups used in monitoring of bioaerosols emitted by composting facilities and their background concentrations.

**Table 3:** **Microbial groups used to monitor bioaerosols from composting facilities from O'Gorman and Fuller (2008), Schlosser et al. (2009), Persoons et al. (2010), Pankhurst et al. (2011), ADEME (2012), Le Goff et al. (2012), Schäfer et al. (2013), and Betelli et al. (2013)**.

**Microbial group**	**Technique**	**Background levels**^a^****	**Concentrations in aerosols from composting facilities**^b^****
		**UFC, gene copies, or cells/m**^c^****	**UFC, gene copies, or cells/m**^3^****
Mesophilic bacteria	Culture	1.6 ± 1.2 × 10^3^ *n* = 13	10^2^–10^8^
Total bacteria	Epifluorescence microscopy (DAPI)	2.5 ± 6.9 × 10^6^ *n* = 16	10^5^ –6.5 × 10^9^
Viable bacteria	Solid-phase cytometry	2.3 ± 1.9 × 10^3^ *n* = 16	9 × 10^4^ –2 × 10^8^
Gram-negative bacteria	Culture		10 –8 × 10^5^
Thermophilic bacteria	Culture	10 – 1.6 × 10^3^	3 × 10^1^ – 10^9^
Thermophilic actinomycetes	Culture		10^2^ – 4 × 10^7^
Molds	Culture	1.1 ± 0.8 × 10^3^ *n* = 13	10^1^ – 10^7^
*Aspergillus* spp.			9 × 10^2^ – 7 × 10^4^
*Aspergillus fumigatus*	Culture	<80	<10^2^ – 4 × 10^7^
*Saccharopolyspora rectivirgula*	qPCR		10^2^ – 1.5 × 10^7^
*Saccharopolyspora rectivirgula* and *rel.*^*c*^	qPCR	1.9 ± 2.3 × 10^3^ *n* = 16	5 × 10^3^ – 4 × 10^7^
NC38, phylotype affiliated to the *Thermoactinomycetaceae*	qPCR	0.9 ± 1.4 × 10^3^ *n* = 16	2 × 10^3^ – 2 × 10^6^
EQ05, phylotype affiliated to *Thermomyces*	qPCR	0.7 ± 1.9 × 10^5^ *n* = 16	10^4^ – 5 × 10^8^
*Thermoactinomyces vulgaris*	qPCR		3 × 10^2^ – 3 × 10^6^

a concentration in air collected in unaffected areas (samples collected upwind or in natural environments).

b concentration in air from composting sites during activities causing bioaerosol emissions; concentrations are expressed as Unit Forming Colonies/m*^3^* for culture, as gene copies/m*^3^* for qPCR, and as cells/m*^3^* for epifluorescence microscopy and cytometry.

c The qPCR system targets partial 16S rDNA sequences from Saccharopolyspora rectivirgula and from phylotypes dominating 16S rDNA molecular inventories in aerosol emitted on composting facilities, and having a close phylogenetic positioning to S. rectivirgula.

An efficient indicator for tracing bioaerosols from composting should have the following characteristics: (i) be readily transposed into an aerosol in high concentrations during the stages of composting that produce bioaerosols; (ii) be specifically associated with the “compost” environment such that it is scarce in the air in environments not associated with composting activities. However, microorganisms such as *A. fumigatus*, *Thermoactinomyces* or *Saccharopolyspora* are not specific to a compost origin (Song et al., 2001; Pankhurst et al., 2011). Indeed, they play an important role in other habitats where decomposition of organic matter takes place at high temperatures and under aerobic conditions (e.g., improperly stored hay, cereal grains, manure, straw, etc.). It is therefore important to analyze other potential source of emissions (agricultural activities) when collecting air samples for dispersal studies.

In the literature, very disparate results can be found concerning the distance at which composting bioaerosols remain detectable. Some authors did not expect finding elevated loads beyond a distance of 150 m from the facilities during normal operation (Reinthaler et al., 1997; Swan et al., 2003). In other studies, however, microbial concentrations fell to the background level only at distances further then 500 m (Hryhorczuk et al., 2001; Recer et al., 2001; Fischer et al., 2008). Le Goff et al. (2012) compiled data obtained from 12 different sampling campaigns carried out at 11 composting plants at distances from 30 to 500 m, with samples collected during a turning activity. For all campaigns, an impact was measureable up to distances of 100 m. Further away, the impact was not systematically observed as it depended on meteorological conditions (wind speed) and on levels of bioaerosol emissions. Beyond 200 m, the signal was largely dispersed, falling to the background level.

The UK Environmental Agency considers that concentrations can return to those of the background noise as near as 250 m from the source emission (Environment Agency, 2001, 2010). However, some studies show the presence of bioaerosols at much greater distances (Recer et al., 2001; Kampfer et al., 2002; Fischer et al., 2008). Fischer et al. (2008) observed that, in normal wind conditions and as a function of the site investigated, the concentrations of thermophilic actinomycetes and of thermotolerant fungi at a distance of 600–1400 m from the site were 1–2 orders of size greater than the background noise. Recer et al. (2001) analyzed the aerosol bio-concentration upstream and downstream of a composting site, with sampling done roughly once a week over a year. The authors concluded that the emissions could increase the level of exposure to bioaerosols up to at least 500 m from the site. Lastly, according to Pankhurst et al. (2011), the reversion to levels measured upstream will not take place at the same distance for each of the different components of the bioaerosol. Actinomycetes and Gram-negative bacteria did not return to upwind levels until 300–400 m downwind, although other bioaerosols (*A. fumigatus*, endotoxins) reduced to concentrations statistically similar to upwind within 250 m from site.

The concentration and composition of bioaerosols at a given point in the environment close to a composting site will depend on many factors. These include (Recer et al., 2001; Jones and Harrison, 2004; Pankhurst et al., 2011): (i) the size and topography of the composting site, (ii) the composting activities in progress and the technology used (which can modify the level of emissions), (iii) the physical/chemical characteristics (humidity, granulometry) of the microflora in the handled compost and (iv) the meteorological conditions (wind speed, temperature, hygrometry, hours of sunshine…). The meteorological conditions are effectively the determining factor for the fate of the particulate material in the atmosphere and, also, for the survival of microbes. Most of the microorganisms caught up in aerosols (with the exception of those having a protective form such as spores) would be rapidly inactivated in air because of the process of desiccation, warm temperatures or UV radiations (Mohr, 1997). It should be noted that the effect of each of these factors remains poorly characterized.

Pankhurst et al. (2012) showed how specific site parameters such as compost process activity and meteorological conditions affect bioaerosol communities, although more data are required to qualify and quantify the causes for these variations. Overall, our understanding as to how the microflora changes in aerosols according to the composting process is limited.

## Using modeling to assess emission flux and dispersal

Models have been used to predict downwind concentrations based on at- or near-source measurements (Swan et al., 2003). Most authors have assumed that bioaerosol spores are sufficiently small to model bioaerosols as a gas and to permit the use of Gaussian dispersion models such as the Pasquill model, the US EPA SCREEN3 and ADMS (Atmospheric Dispersion Modeling System) (Drew et al., 2006). The literature on modeling the dispersal of bioaerosols emitted by composting facilities is not abundant. This is partly due to the fact that a facility's source term is difficult to calculate. Activities will produce episodic or periodic releases of aerosols due to factors such as operational cycles, fluctuations in the daytime temperature that alter the characteritics of the emissions, or fluctuations in atmospheric pressure that dictate the initial release of pollutants. Furthermore, given the range of activities (shredding, screening, turning, moving the windrows…) there are often a number of sources which make up a “source term” (Taha et al., 2006). Taha et al. (2006, 2007) used source depletion curves drawn up for *A. fumigatus* and actinomycetes during composting activities (turning, shredding, screening) to estimate emission rates and then evaluated the distance at which concentrations fell to background levels using SCREEN 3. They showed that bioaerosol concentrations are likely to decrease to within acceptable levels before the UK Environment Agency 250 m risk assessment threshold. Some rare studies have combined bioaerosol dispersion modeling results with models calculating human exposure (Dowd et al., 2000; Chalvatzaki et al., 2012). Chalvatzaki et al. (2012) analyzed the effect of dust emissions from open storage piles at a municipal solid waste composting site and concluded that the exposure to PM_10_ for an adult who is not working at the composting site was 20–74% lower compared to that of a worker at the composting site.

## Prospects for the future

The impact of composting facilities on air quality in downwind environments remains difficult to assess. In particular, the distance at which the bioaerosol concentration reverts to the level of the background noise is still under debate. The different results in the literature are due notably to the variable nature of emissions as well as to the influence of diverse factors on aerosol dispersal. Modeling studies can help to better assess bioaerosol dispersal and facilitate conclusions concerning risk assessment. Molecular techniques provide access to non-culturable microorganisms and are widely used to monitor microorganisms in water or soil. Integrating data obtained using molecular techniques into modeling should enhance understanding of dispersal of bioaerosols. Today, several microbial indicators with good specificity to compost origin are available which can be monitored by qPCR. Combining molecular tools and modeling constitutes one important future line of investigation.

When modeling dispersion, particle size and agglomeration play an important role in the aerodynamics of bioaerosols. Furthermore, these factors determine the penetration into the human respiratory system. Additional field studies are required to examine particle size distribution in bioaerosols emitted by composting facilities along with the possible tendency of bioaerosols to form aggregates.

Furthermore, the study carried out by Pankhurst et al. (2011) showed differences in the dispersion of *A. fumigatus*, the actinomycetes and Gram-negative bacteria. This can be explained by the fact that the ecology of the micro-organisms, their physiology and their mechanisms of dissemination (sporulating and non-sporulating microorganisms) all influence the formation of aerosols and their dispersion in the atmosphere. Thus, it is important to gather more data on the emission rates and the dispersal of the indicators used to trace the aerosols emitted by composting facilities, and, also, to compare them to the other microbial components of the aerosols.

The changes in the microbial make-up of the aerosols emitted at the different stages of the composting process must be better characterized, in light of the microbial diversity of the source, i.e., the compost. This would help us to understand the mechanisms of selection during aerosol emission, insofar as some microorganisms are more prone to being aerosolized. Diversity studies could also help in identifying the microbial agents responsible for effects on health.

More research is needed on analyzing the emission and dispersal of bioaerosols emitted by composting facilities in order to better implement regulations by determining acceptable levels of bioaerosols and defining buffer zones between compost sites and nearby residential areas. Regulations should evolve together with monitoring techniques and take into account recent advances in molecular tools.

### Conflict of interest statement

The author declares that the research was conducted in the absence of any commercial or financial relationships that could be construed as a potential conflict of interest.
